# Evaluating risk detection methods to uncover ontogenic-mediated adverse drug effect mechanisms in children

**DOI:** 10.1186/s13040-021-00264-9

**Published:** 2021-07-22

**Authors:** Nicholas P. Giangreco, Nicholas P. Tatonetti

**Affiliations:** Departments of Systems Biology and Biomedical Informatics, Columbia University, 622 W. 168th Street, New York, NY 10032 USA

**Keywords:** Pharmacovigilance, Pediatrics, Child development, Modeling, Dynamics

## Abstract

**Background:**

Identifying adverse drugs effects (ADEs) in children, overall and within pediatric age groups, is essential for preventing disability and death from marketed drugs. At the same time, however, detection is challenging due to dynamic biological processes during growth and maturation, called ontogeny, that alter pharmacokinetics and pharmacodynamics. As a result, methodologies in pediatric drug safety have been limited to event surveillance and have not focused on investigating adverse event mechanisms. There is an opportunity to identify drug event patterns within observational databases for evaluating ontogenic-mediated adverse event mechanisms. The first step of which is to establish statistical models that can identify temporal trends of adverse effects across childhood.

**Results:**

Using simulation, we evaluated a population stratification method (the proportional reporting ratio or PRR) and a population modeling method (the generalized additive model or GAM) to identify and quantify ADE risk at varying reporting rates and dynamics. We found that GAMs showed improved performance over the PRR in detecting dynamic drug event reporting across child development stages. Moreover, GAMs exhibited normally distributed and robust ADE risk estimation at all development stages by sharing information across child development stages.

**Conclusions:**

Our study underscores the opportunity for using population modeling techniques, which leverage drug event reporting across development stages, as biologically-inspired detection methods for evaluating ontogenic mechanisms.

**Supplementary Information:**

The online version contains supplementary material available at 10.1186/s13040-021-00264-9.

## Background

Adverse drug events (ADEs) in children are common and can result in injury and death [1, 2]. Clinical trials rarely include children [3] and pediatric-specific trials are limited in identifying possible ADEs in the population [4]. Pediatric drug safety studies can evaluate large numbers of ADEs from the population [5] but current methodologies are limited in their ability to identify the mechanisms that drive pediatric ADEs [6]. Children undergo evolutionarily conserved and physiologically dynamic biological processes, collectively called ontogeny, as they grow and develop from birth through adolescence [7, 8]. The mechanisms may include varying protein activity [9, 10] as well as include functional and structural changes that occur during maturation [11, 12]. These ontogenic changes can alter pharmacodynamics and pharmacokinetics resulting in adverse effects, as is the case for doxorubicin-induced cardiotoxicity [13] and valproate-induced hepatotoxicity [14]. With a few notable exceptions, however, many pediatric adverse events are idiopathic with no known, clear connection to developmental biology [15, 16]. Additionally, adverse event mechanisms established in adults may not translate to the pediatric population [17]. There is an opportunity to combine known ontogenic biology with real-world pediatric drug effect data to identify ontogenic-mediated adverse events.

To date, elucidation of ontogenic mechanisms has relied on hypothesis-driven approaches. For example, juvenile mouse models have been used to identify genetic vulnerabilities of hematopoiesis [18] and investigate effects by a glutamatergic agonist on the neural developmental sequence [19] during early life. More recently, pharmacometric tools have been used to extrapolate drug effects from adults to children, such as projecting acetaminophen exposure across pediatric age groups [20], and investigate drug action in children, such as predicting clearance of zidovudine during infancy [21]. However, juvenile animal studies are low-throughput and require complex study designs [22], and there is limited experimental data to parameterize manually designed pharmacometric models [23, 24]. While lacking specificity, top-down studies are complementary in that they evaluate thousands of hypotheses simultaneously and can identify idiosyncratic effects that would otherwise go unnoticed [25, 26]. Moreover, analyses of large population datasets start from clinically significant events which can take decades to identify [27, 28]. Top-down studies can close the pediatric evidence gap [23] by sifting through large databases to identify clinically significant although perhaps less studied and rare adverse drug events during the period of child growth and development.

While pediatric pharmacovigilance has been able to identify adverse drug events, it is limited in identifying growth and development processes that underlie those observations [10, 29]. A common approach when identifying ADEs is to stratify the pediatric population into age groups. The Proportional Reporting Ratio, which was designed to be sensitive even when data is scarce [30], is an established detection method and has been shown to unmask ADE signal within child development stages compared to detection within the larger pediatric population [31]. However, this common practice in pediatric drug safety directly reduces the amount of data available to identify ADEs during childhood. Reduced data within these strata was shown to significantly affect PRR detection performance across pediatric age groups [31]. In contrast to population stratification, the continuous, time-dependent biological processes during growth and development suggest using all information across child development stages to identify pediatric ADEs.

Generalized additive models (GAMs) are supervised machine learning approaches that can quantify non-linear effects reflective of natural phenomena [32]. GAMs may be able to quantify signal reflecting dynamic, continuous processes such as ontogeny. These models are extensively used for spatial and temporal analysis in ecological studies [33], such as explaining cardiovascular mortality risk from heat waves over time [34] and rat infestation from environmental factors within geographic areas [35]. Similar to evaluating ecological responses using shared information across time or space, we can evaluate adverse events from temporally-connected ontogenic processes using shared information across child development stages.

Our study evaluates the novel application of the GAM in modeling age groups in the pediatric population to detect co-occurring and possibly dynamic adverse drug event reporting across childhood. We performed a data simulation and augmentation study that 1) simulated drug event reporting temporal trends of different effect sizes and shapes, 2) augmented existing pediatric drug event data by inserting the simulated reporting rates within observational data, and 3) evaluated population stratification (PRR) and modeling (GAM) methods to detect these injected ADE reporting dynamics. We found the detection scores generated by the GAM showed improved risk estimates and increased detection of drug event reporting among the various simulated dynamics compared to the PRR. Detection methods that capture temporal adverse drug event dynamics within observational databases can improve our understanding of the interactions between child developmental biology and adverse drug effects.

## Results

### Pediatric FAERS

**Fig. 1:**
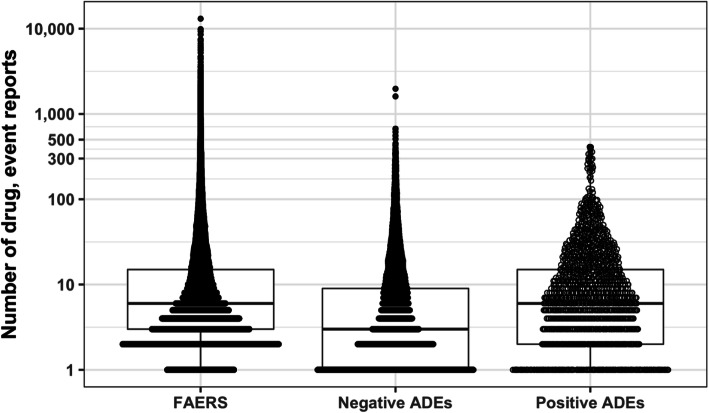
Comparison of drug event reporting in drug-event datasets. A boxplot summary overlayed by the amount of drug event reports for drug-event pairs between (pediatric) FAERS (*N* = 519,555), the positive control set (*N* = 500), and the negative control set (*N* = 10,000). ‘N’ is the sample size

Our study evaluates population stratification versus modeling statistical methods to detect co-occurring adverse events and drugs in the FDA Adverse Event Reporting System (FAERS). There were 339,741 pediatric drug event reports in FAERS, which contained 519,555 unique drug-event pairs. We constructed positive and negative sets of drug-events to evaluate detection of putative dynamic drug event reporting. We randomly sampled 500 drug-event pairs to be augmented with simulated drug event reporting dynamics, representing our positive control set for drug-events with putative dynamics. We then randomly sampled another 10,000 complementary drug-event pairs where the underlying data was untouched, representing our negative control set of drug-events with no known dynamics. We showed there was no significant difference in the amount of drug-event reporting between FAERS and the negative control (2-sample Student t-test *p*-value = 0.98) or positive control (*p*-value = 0.79) drug-event pairs (Fig. 1).

### Data simulation and augmentation

**Fig. 2:**
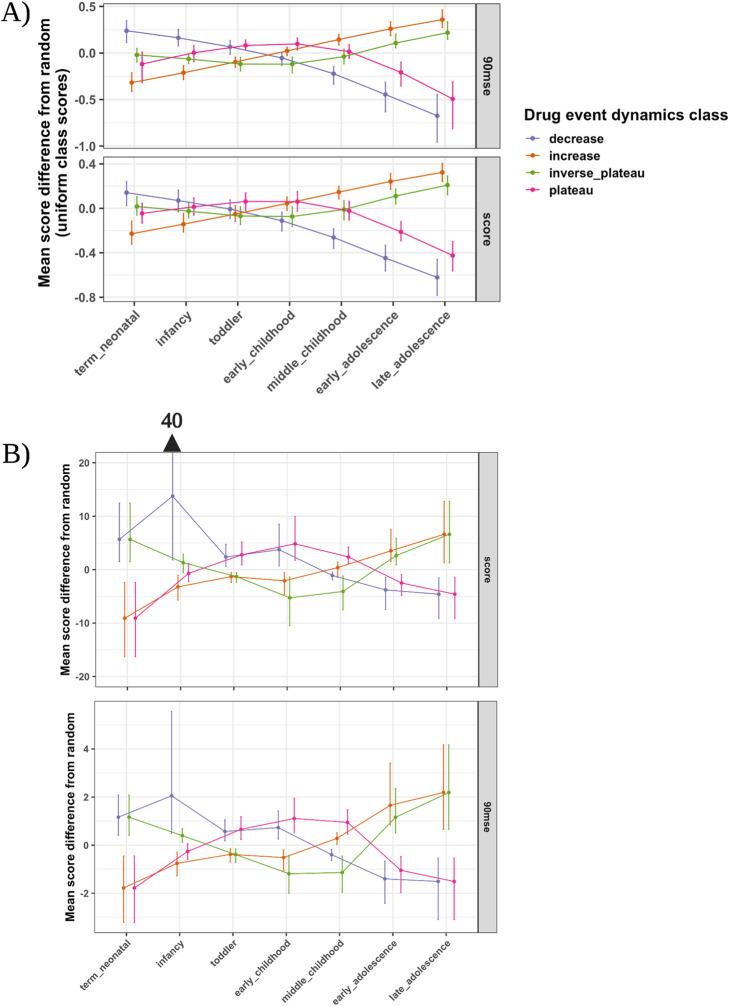
ADE detection method risk score distribution across child development stages. Risk scores resulting from applying the **A** GAM and **B** PRR ADE detection methods on the positive control drug-event pair data of each dynamics class. The score distributions at each child development stage were produced after 100 bootstraps of the original scores for each method and score type. We show the average difference of the resampled score distributions between a given drug event reporting dynamics class and uniform (random drug event reporting across childhood) with the 95% confidence interval

We augmented the 500 drug-event pairs in the positive control set with putative drug event dynamics (see Methods). Augmenting the positive control data with drug event reporting dynamics did not have a systematic effect on the amount of drug event reporting compared to the untouched negative control set (Figure S1). However, applying the PRR and GAM detection methods onto the positive control data showed the generated risk scores reflected the simulated dynamics classes (Fig. 2).

**Fig. 3:**
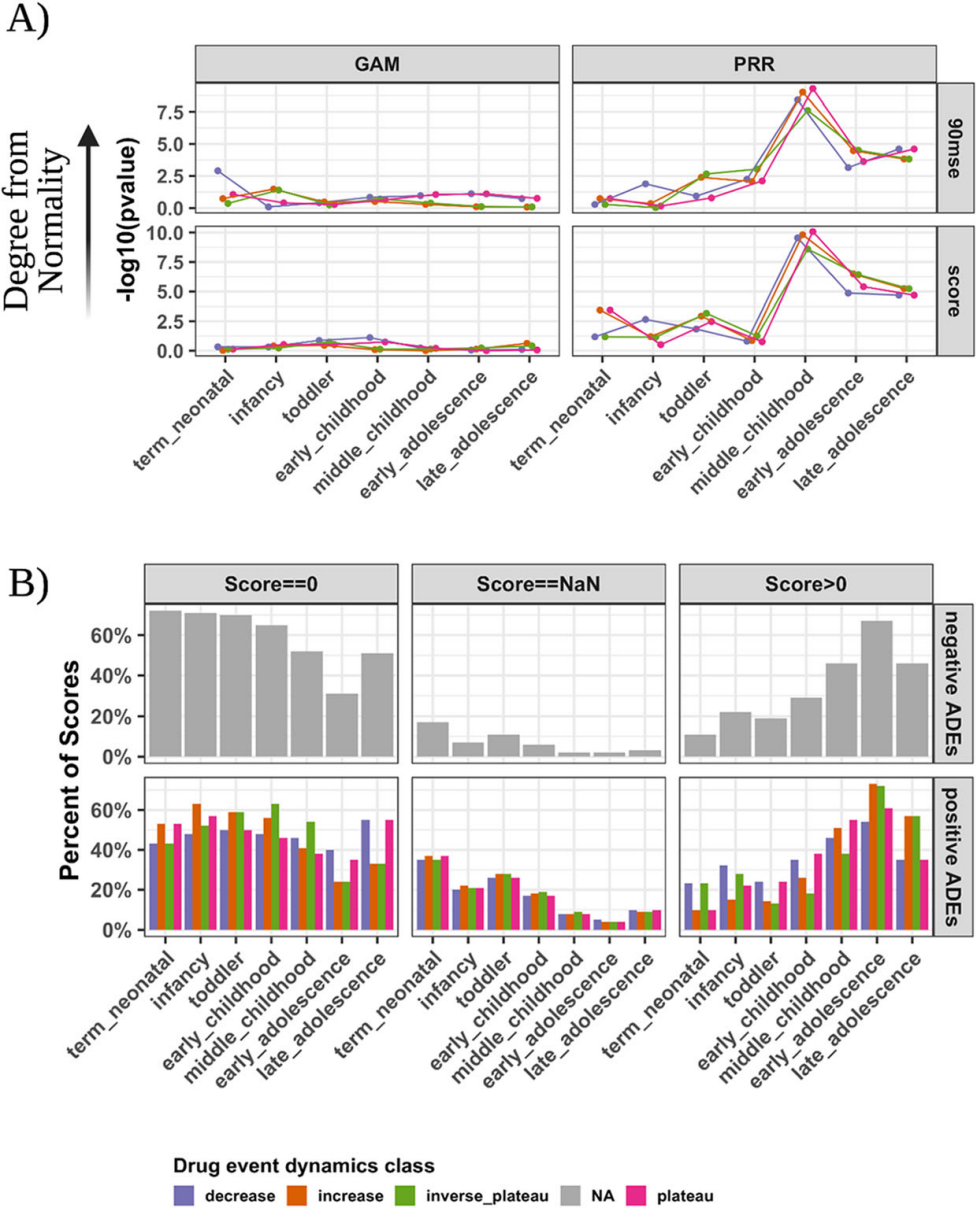
Summary of ADE detection method risk score quality. **A** Deviation from the normal score distributions for each method and score type across development stages. The score distributions were produced after 100 bootstraps of the original scores at each child development stage. The Shapiro-Wilk test calculated a significance probability value for the resampled scores being drawn from a normal distributions. **B** PRR detection method risk score quality summary. Across the positive and negative controls as well as drug event reporting dynamics classes, we calculated the number of scores with a zero, NaN (unable to be computed; the drug not reported at a stage or the drug not reported with the event at a stage), or nonzero positive score across child development stages

We evaluated the drug event risk scores of each detection method in reflecting the putative drug event reporting dynamics. The GAM generated all finite and nonzero ADE risk that resembled normally distributed scores (Shapiro-Wilk test average *p*-value and 95% confidence interval: 0.56 [0.12, 0.98], 90mse: 0.34 [0.023, 0.83]) in comparison to the PRR (score: 0.04 [1.29E-10, 0.22], 90mse: 0.14 [8.06E-10, 0.78]) at child development stages (Fig. 3A). Moreover, 47% of PRR scores were zero and 18% were unable to be computed, on average, for drug-event pairs (Fig. 3B).

### ADE dynamics detection performance

**Fig. 4:**
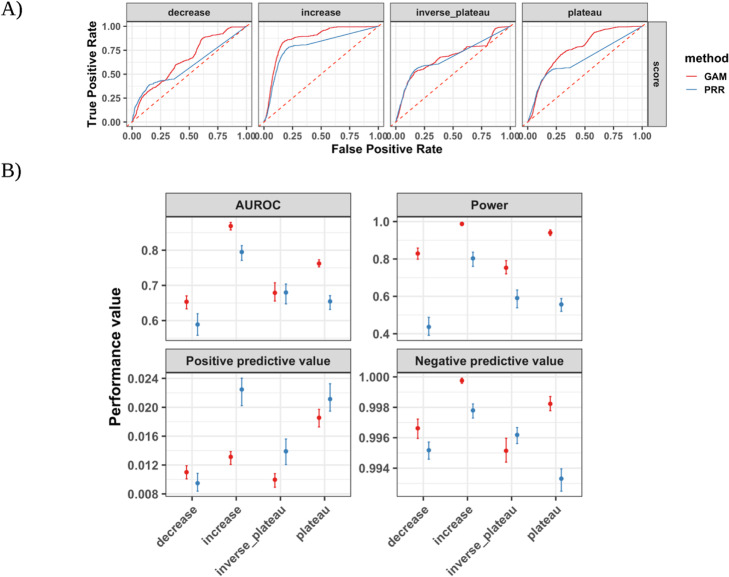
GAM and PRR drug event dynamic detection score performance. **A** The receiver operating characteristic curves showing the true positive rate versus the false positive rate for each method by drug event reporting dynamics class. The **B** area under the receiver operating characteristic curve (AUROC), power (i.e. sensitivity or true positive rate, positive predictive value (i.e. precision), and negative predictive value across child development stages for each dynamic class

**Fig. 5:**
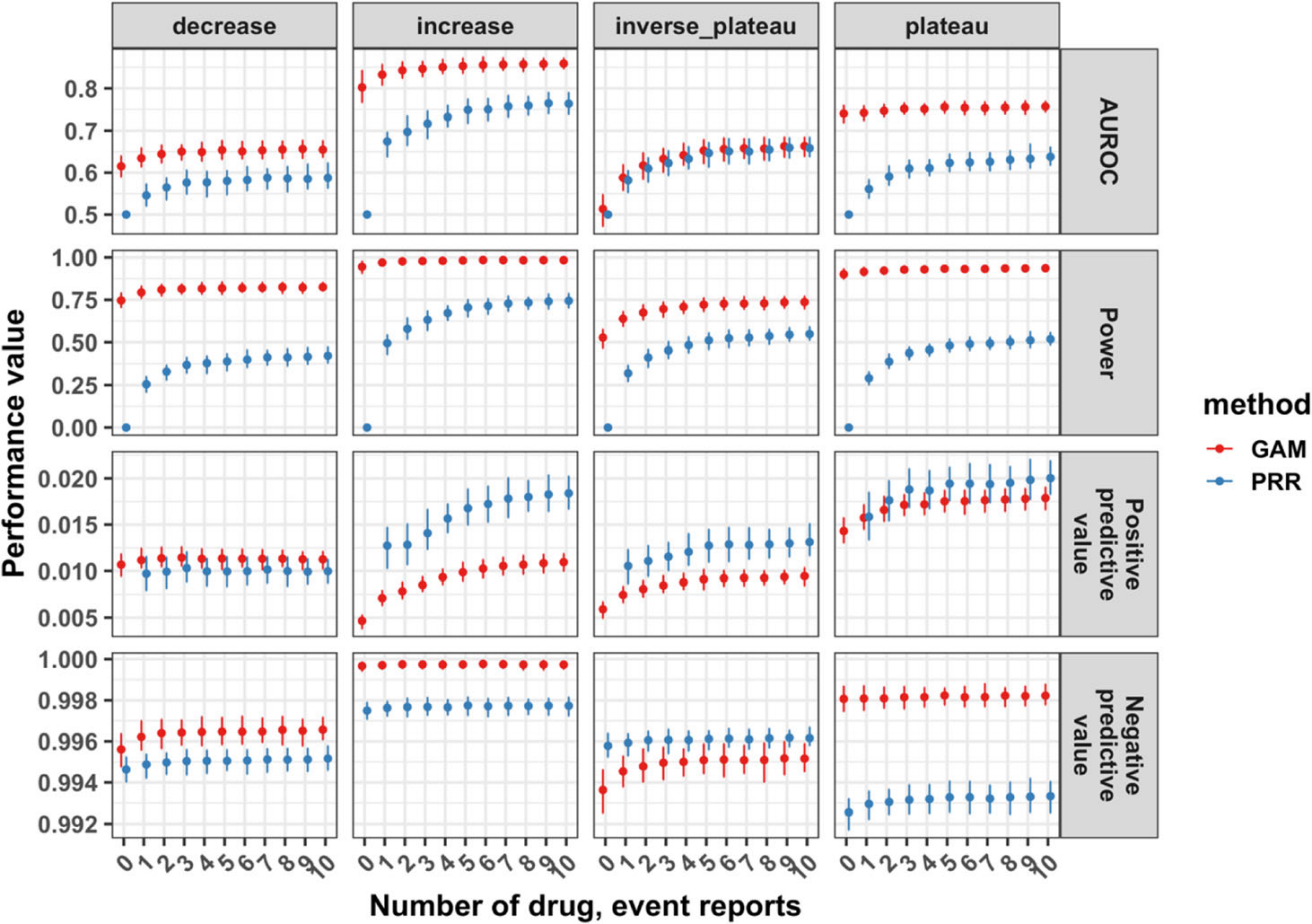
GAM and PRR drug event dynamic detection score performance at low drug reporting. The area under the receiver operator characteristic (AUROC) curve, the True positive rate (i.e. power) or sensitivity, the positive predictive value (i.e precision), and the negative preedective value for with a given amount of drug reports

We compared the performance of the GAM and PRR for detecting drug event reporting dynamics. Additionally, we further investigated the performance contribution at each child development stage within the dynamics class. We found that the GAM had higher AUROC and power across stages (Fig. 4 and Figure S2) and similar performance within each child development stage (Figure S3, 4) when detecting drug event reporting dynamics compared to the PRR. When considering low reporting of drug-events, the GAM had substantially higher AUROC and power, at the expense of excess false positives, compared to the sensitive-by-design PRR (Fig. 5 and Figure S5).

### ADE dynamics sensitivity analysis

**Fig. 6:**
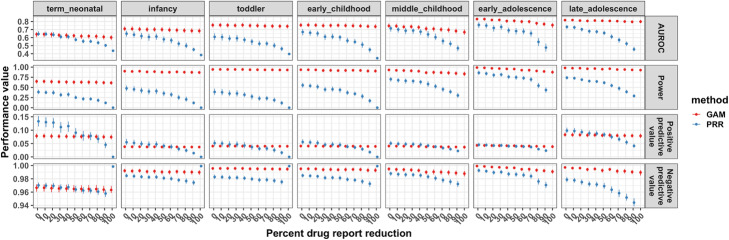
GAM and PRR drug event dynamic detection score performance as drug reports become more rare. The drug reporting drug-event pairs was reduced at 10% decrements only within a specific child development stage. For example, 0% drug reporting reduction indicates no reduction in drug reporting and 100% drug reporting reduction indicates all drug reports were removed. The area under the receiver operating characteristic (AUROC) curve, the power (i.e. true positive rate or sensitivity), the positive predictive value (i.e. precision), and the negative predictive value as drug reports become rare at child development stages

We investigated the detection of drug event reporting across all dynamics while removing drug reports within child development stages (Figure S6 and see Methods). The ADE risk scores generated by the GAM showed dependent, flexible risk estimates across child development stages unlike the PRR (Figure S7). We found that the GAM had more robust and higher overall performance and sensitivity (Fig. 6 and Figure S8) to detect the various drug event reporting dynamics as adverse drug events became rare at child development stages.

### Real-world validation

**Fig. 7:**
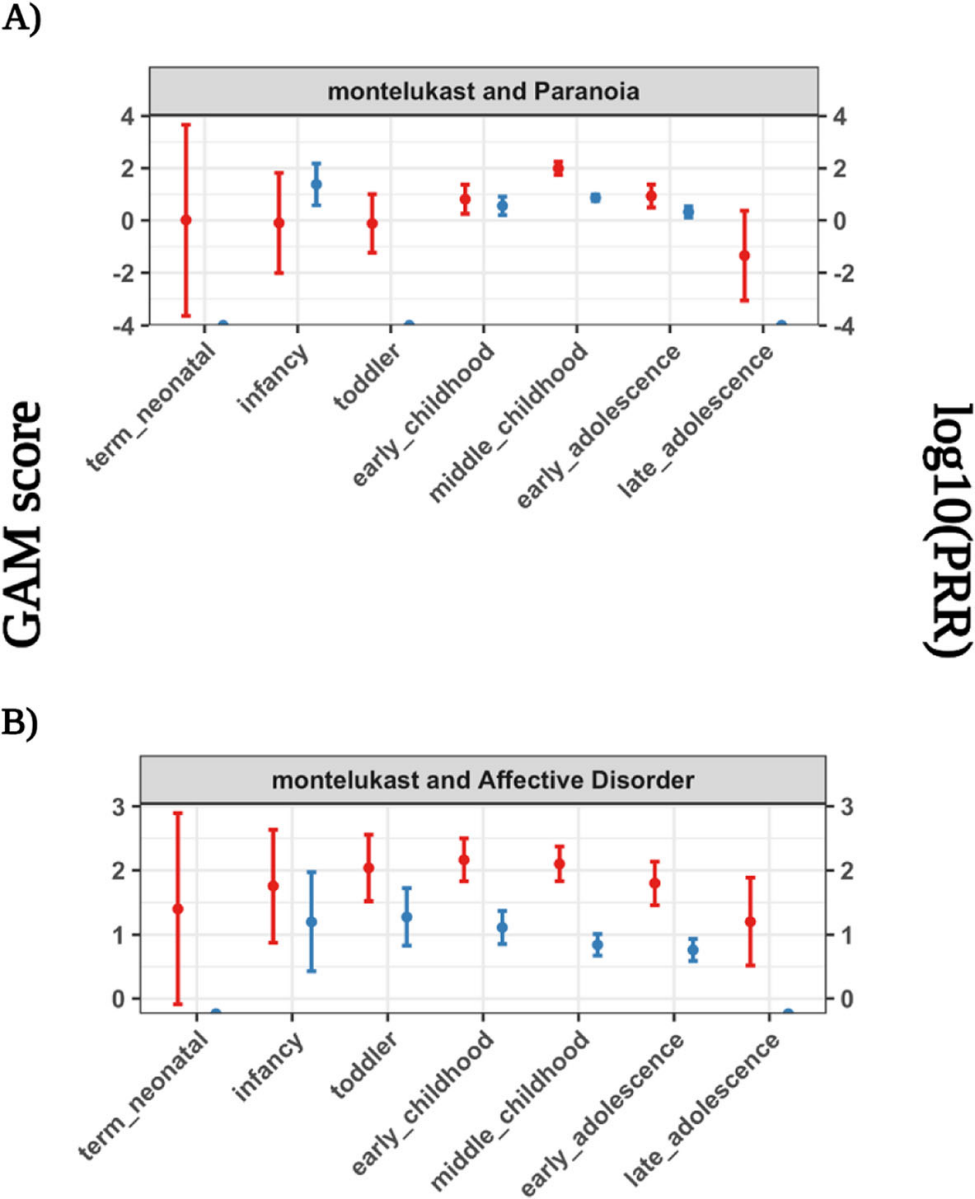
GAM and PRR detection scores on putative real-world dynamic adverse drug events. We highlight two psychiatric adverse events, **A** Paranoia and **B** Affective Disorder, from exposure to the drug montelukast exhibiting dynamic ADE risks across child development stages

We conducted real-world validation to evaluate ADE risk detection against a manually curated reference set of pediatric adverse drug events. We compared the performance of the GAM and PRR for detecting drug-event pairs in a real-world pediatric reference set of 26 drug-event pairs (see Methods and Figure S9). We found that the GAM had slightly improved overall performance and sensitivity compared to the PRR for detecting pediatric adverse drug events (Table 1 and Figure S10). Moreover, we found no difference in the fraction of drug-event pairs with significant ADE risk at child development stages (Table 2; proportion test *p*-value = 0.39). We found that the GAM identified two real-world pediatric drug events with putative dynamic ADE risk (Fig. 7). Specifically, the GAM showed periods of lower risk during early and late childhood and higher risk during the middle stages of childhood. While the PRR and GAM performed approximately the same overall, the GAM captured dynamic ADE risk where the PRR did not.

**Table 1:** Real-world pediatric drug-event detection performance. The area under the receiver operating characteristic curve (AUROC) and sensitivity or true positive rate to detect real-world pediatric drug-events observed within FAERS per each method and score type. The prediction threshold for the sensitivity was the null statistic for each method (null threshold: GAM==0; PRR==1). The performance interval is the 95% confidence interval

	Real-world pediatric drug-event detection performance							
	AUROC				Sensitivity			
	90mse		score		90mse		score	
	PRR	GAM	PRR	GAM	PRR	GAM	PRR	GAM
Performance value	0.62 [0.58, 0.66]	0.65 [0.59, 0.69]	0.62 [0.58, 0.67]	0.73 [0.69, 0.77]	0.28 [0.22, 0.36]	0.28 [0.23, 0.35]	0.49 [0.40, 0.56]	0.85 [0.81, 0.89]

**Table 2:** ADE detection method risk score quality and significance on real-world pediatric drug-events. The number of drug-event pairs that contained a child development stage with a risk score of each score quality (null threshold: GAM==0; PRR==1). A 90% lower bound score above the null threshold indicates a significant risk.

Number of drug-event pairs with score quality in atleast one stage	Zero	NaN	Finite	Infinite	Score above null	90% lower bound above null
GAM	0	0	26	0	26	14
PRR	23	14	25	5	26	18

## Discussion

We present the first study to evaluate methods to detect drug event reporting patterns across childhood in large observational data. Children undergo a period of dynamic growth and development, presenting a challenge in identifying and evaluating adverse drug events [10, 36]. We hypothesize that dynamic ontogenic processes as children grow and develop may be reflected by temporal drug event reporting in the population. We found that GAMs, a population modeling technique, outperformed the PRR, a population stratification method, as well as generated robust risk scores to detect adverse drug events during childhood. This work represents a first step in transitioning from performing event surveillance towards uncovering putative mechanisms of pediatric adverse drug events.

The goal of our study is to improve the specificity of top-down data mining for generating pediatric drug safety hypotheses. We hypothesized that temporal drug event reporting trends found in observational data are dependent on ontogeny, which exhibits high and low molecular and physiological levels throughout childhood [7, 37, 38]. To test this within a top-down approach, we generated temporal trends in observational data to correspond with putative temporal trends from ontogeny as opposed to identifying temporal trends from confounded frequency [39] or feature-derived [40, 41] measures. This motivated both simulating dynamic drug event reporting rates and then augmenting real-world data to generate different classes of dynamic drug event reporting trends. While we simulated dynamic drug event reporting rates, we showed that augmenting the FAERS data did not change the overall characteristics of the pediatric drug reports. This was crucial for establishing the use of real-world drug event data to evaluate hidden dynamic reporting trends. Importantly the ADE detection methods were in fact able to identify the simulated dynamics within the data. The data simulation and augmentation of FAERS laid the foundation for evaluating statistical methods to investigate ontogenic-mediated adverse event mechanisms.

We found that the generalized additive model (GAM) showed improved detection of dynamic drug event reporting compared to the proportional reporting ratio (PRR). While the PRR produced ADE risk scores that were more erratic and unable to be computed, the GAM scores were both more flexible and robust. The GAM assumes a flexible relationship yet reduces ‘wiggliness’ to stable risk estimates based on observed data [42, 43]. While bayesian modeling techniques such as Monte Carlo Markov Chain can also learn flexible relationships from observed data, these models still require expert knowledge to build, implement, and interpret [44]. The GAM, on the other hand, generates an interpretable smooth relationship in a familiar regression framework [32] that shares information across child development stages. Using this shared information framework, the GAM was able to detect injected dynamic ADE risks across childhood even when drug event reporting was low. We further showed that the GAM not only generated visually dynamic ADE risk when injecting dynamics (Figure S11A), but we also identified putative dynamic risk for real-world psychiatric adverse events from exposure to montelukast medication (Figure S11B). We demonstrated that GAMs can be used to detect dynamic reporting of adverse drug events by sharing information across child development stages.

Our study highlighted when sharing information resulted in the GAM performing well as well as the methods limitations. We found that detecting drug event report dynamics overall was at least similar if not improved for risk scores, with slightly reduced advantages by the more conservative 90% lower bound scores. Interestingly, at stages that did not have reports of a drug and event co-occurring, the GAM showed substantial power to detect dynamics using information from adjacent stages. However, increased detection often came at the expense of higher false positives for the GAM especially at low reporting. Moreover, removing drug reports significantly reduced the ability of the PRR to detect dynamics with larger uncertainty compared to the GAM (Figure S12). Overall, we argue the benefits afforded by the GAM, namely robust, flexible risk estimates at small data and increased sensitivity, presents an opportunity for biologically-inspired data mining for detecting often rare pediatric adverse events.

This study has limitations. First, observational data has inherent bias and confounding factors which may affect both the sample of drug-event pairs in our study as well as the performance of the detection methods. We showed that the random sample of drug events correspond to the reporting patterns found in the FAERS database. Also, performing a power analysis allowed for identifying drug events for which the detection methods were able to identify the dynamic reporting to provide a fair performance comparison. Second, other regulatory agencies, such as the Food and Drug Administration and European Medicines Agency, define pediatric age ranges for development stages by different methods. While varying child stage definitions were not explored here, we chose stages defined by NICHD that were established after consultation and agreement among several US-based organizations such as the American Academy of Pediatrics and the Centers for Disease Control and Prevention [45]. Third, fixed development stages may serve more useful in drug regulations and trial design than representing dynamic child growth and development. Nevertheless, the detection performance and risk scores for both methods could only be compared when considering data found within child development strata. Fortunately, the further advantage of the GAM is its ability to model childhood as a continuous period using age without restrictive strata. This increases the sharing of information for identifying adverse event risk during childhood which may cross development stages and affect specific periods during childhood. This approach to share information becomes less powerful as the noise-to-signal content increases.

## Conclusion

In this study, we evaluated ADE risk detection methods to identify dynamic drug event reporting within observational data. By simulating drug event reporting and augmenting simulated rates into existing observational data, we can make comparisons between methods to detect dynamic drug event reporting patterns. We found GAMs result in more robust scores, overall improved performance to detect dynamics, and improved ability to detect simulated and real-world pediatric drug-events compared to the state-of-the-art in pediatric drug safety studies. This study lays the foundation to detect and evaluate pediatric adverse drug events for ontogenic-mediated mechanisms.

## Methods

### ADE data source

We retrieved drug event reports from the Food and Drug Administration’s openFDA [46] download page, utilizing an API key with extended permissions, containing the FAERS data. Using custom python notebooks and scripts available in the ‘openFDA_drug_event-parsing’ github repository (DOI: 10.5281/zenodo.4464544), we extracted and formatted all drug event reports prior to the third quarter of 2019. Data fields included the safety report identifier, age value, age code e.g. year, adverse event MedDRA concept code (preferred terms), and drug RxNorm code (various) used in our analyses. The age value was standardized to year units for categorizing reports into the 7 child development stages according to the Eunice Kennedy Shriver National Institute of Child and Human Development [45]. Adverse drug event MedDRA codes were mapped to standard concept identifiers using concept tables [47] from the OMOP common data model. The drug RxNorm code was similarly translated to the standard RxNorm concept identifier (ingredient level) in OMOP and was further mapped to the equivalent ATC concept identifier (ATC 5th level) using the concept relationship table. The occurrence of an adverse drug event is defined as any safety report where both the adverse event and drug concepts are reported together. The pediatric report space for any adverse drug event is all reports which have age above zero and less than or equal to 21 years old which is the upper bound for the late adolescence child development stage. The drug event data for a given drug-event pair composed of 339,741 safety reports with a binary indicator for reports of the event and drug, as well as the category of NICHD child development stage for the report’s patient.

### Simulated ADE dynamics

The objective of this study was to evaluate detection of drug-event reporting as the reporting rate changes across child development stages with varying dynamics and effect sizes. We assert that reporting dynamics during childhood reflect ontogenic profiles observed on molecular, functional, and structural levels [7, 37, 38].

We simulated dynamic ADE reporting by combining hyperbolic tangent functions that produced symmetric probability distributions around a given effect size to define the probability of event reporting at drug reports. These dynamic reporting classes represent nonlinear trends of drug-event reports across childhood. The average drug and event reporting across reports equaled the event reporting rate multiplied by a fold change factor resulting in the effect size of dynamic drug event reporting. The fold change followed a negative exponential distribution with rate parameter 0.75 resulting in a fold change distribution ranging from 1 to 10 (Figure S13). The simulated reporting probabilities set the Bernoulli random variable to assign the presence (1) or absence (0) of the event being reported for each of the 339,741 safety reports. We designed 5 different dynamic reporting rates, namely ‘uniform’ (random), ‘increase’, ‘decrease’, ‘plateau’, and ‘inverse_plateau’ (Figure S14).

### ADE data augmentation

We augmented the original drug event data from FAERS with the simulated drug event reporting dynamics. We randomly selected 500 drug-event pairs to be the positive control set for drug-events with putative drug event reporting dynamics. We augmented the drug event data for each pair with the previously described dynamics that we want to detect. We then randomly selected 10,000 mutually exclusive drug-event pairs to be the negative control set which were not augmented and represented observed reporting of drugs with events within FAERS. Differences of the average drug event reporting between the drug-event sets was computed by comparing 10 million resamples of each distribution.

Augmenting the positive control drug-event pairs resulted in 5 sets of 500 drug-event pairs, forming (drug-event, stage, dynamic) triples. The (drug-event, stage, uniform dynamic) triple scores were the reference distribution for comparing the average difference in scores, after 20 resamples, with ADE risk scores from the other dynamics classes.

### ADE detection methods

Our study evaluates population stratification versus modeling statistical methods to detect co-occurring adverse events and drugs in observational data. The proportional reporting ratio (PRR) stratified the pediatric population into child development stages to quantify the odds for event reporting prevalence with a drug compared to without a drug reported. The logistic generalized additive model (GAM) quantifies the log odds for event reporting with the drug compared to without the drug across child development stages. The methods are applied onto the drug event data to evaluate adverse drug event detection in the presence versus the absence of putative drug event reporting dynamics.

We employed the Proportional Reporting Ratio (PRR):

$$ \frac{\frac{a}{a+c}}{\frac{b}{b+d}} $$

where ‘a’ is the number of reports with the drug and event, ‘b’ is the number of reports without the drug and with the event, ‘c’ is the number of reports with the drug and without the event, and ‘d’ is the number of reports without the drug or event of interest. These are the four parameters of the PRR equation. For each drug-event pair, we applied the PRR to each of the 7 child development stages resulting in 7 scores. The PRR scores were log10 transformed when conducting the Shapiro-Wilk test for normality.

We also evaluated the logistic generalized additive model [48] (GAM):

$$ g\left(E(Event)\right)=s\left( nichd, by= Drug\right) $$

where *g* is a logit link function, *E*(*Event*) is the expected value of event reporting, *s* is a spline function with a penalized cubic basis, *nichd* is the child development stage of the report’s subject, and *Drug* is an indicator i.e. 0 or 1 of drug reporting. Details for GAMs can be found at references [42, 49] and we specified the model using the *mgcv* package in R.

Briefly, the GAM is a flexible statistical model that captures nonlinear effects of covariates onto a response. In this paper, we model the effect of the child development stage interacting with drug reporting on the reporting of an event where the event is the reporting of the MedDRA preferred term and the drug is the reporting of the ATC 5th level drug concept. The *s*() function is a spline function where the interaction of the child development stage (main effect) and the drug (interaction using the ‘by’ variable) is modeled according to a set of basis functions. Each development stage defines the knot (7 in total) in which the expectation of event reporting is quantified. In the spline function, a penalized cubic spline basis (bs = ‘cs’) is used for fitting the basis functions where the first and second derivative of the event expectation is zero at each knot, resulting in a smooth event expectation across stages. To mitigate overfitting or ‘wiggliness’, we used a penalized iterative restricted likelihood approach, called ‘fREML’, with a wiggliness penalty in the objective function. Fitting the GAM model (using the ‘bam’ function and discrete = T) produces coefficient terms, similar to beta coefficients in logistic regression, for each child development stage. This produces a model with 8 parameters, one for each coefficient and intercept term (the intercept term is not utilized in this study). For each drug-event, GAM scores are generated for each child development stage resulting in 7 scores. It is important to note that all GAM scores produced are finite, nonzero values.

The scores generated by each method have different variations and uncertainty in the estimated population value. We additionally determined the lower confidence bound in which the population-based score would be greater than 90% of score replicates. The population score and the 90% lower confidence bound, called ‘score’ and ‘90mse’ respectively, are the score types for each method.

### ADE dynamic detection power analysis

We performed a power analysis to determine which of the positive control drug-event pairs could be detected for each method and score type. The generated scores may not show a drug and event association (score above the null statistic or another significance threshold) for a child development stage due to the method’s different assumptions and biases when applied onto observational data. To mitigate these issues, we determined the drug event data characteristics, namely the number of drug reports and the effect size, for each method in which reporting dynamics could be detected at or above *t* = 80% power (the power or true positive rate is the fraction of scores above a number of drug reports and effect size out of all scores; Figure S15). Specifically, for the (drug-event, stage, dynamic) triple scores in the positive control set, we determined the power to differentiate scores at high reporting rates about a given score threshold (GAM score threshold==0; PRR score threshold==1). The reporting rates were higher at different child development stages for each dynamics class e.g. the ‘increase’ dynamics class had higher reporting at the ‘early_adolescence’ and ‘late_adolescence’ stages (Table S1). The scores from (drug-event, stage, dynamic) triples with a high reporting rate were only considered for reflecting dynamic drug event reporting associations. The scores from (drug-event, stage, dynamic) triples with a low reporting rate were not considered further due to spurious scores generated at stages without injected signal. The drug event characteristics were determined for both the estimated population score (‘score’) and the 90% lower bound score (‘90mse’) that represent scores with lower and higher confidence, respectively, for the ‘true’ population score.

Choosing drug-event pairs at or exceeding the characteristics for each method and score type at or above *t* = 80% power resulted in a superset of (drug-event, stage, dynamic) triples designated as positives in a reference standard for each drug event reporting dynamics class (Table S2). The negative control set contained the same (drug-event, stage) doubles or 70,000 scores for each reference standard. Excluding the drug-event scores generated by the uniform class, there were 4 reference standards of positive and negative drug-event pairs for each ADE reporting dynamics class used for detection performance evaluation.

### ADE dynamic detection performance

We evaluated the GAM and PRR methods to detect drug event reporting dynamics across the child development stages. Specifically, we determined the performance in differentiating scores from (drug-event, stage, dynamic) triples in the positive control set versus the negative control (drug-event, stage) score doubles. The positive control set contained a superset of the 500 (drug-event, stage, dynamic) score triples (Table S2). The negative control set contained the same (drug-event, stage) doubles or 70,000 scores for each reference standard. For each of the four reference standards, we quantified the area under the receiver operating characteristic (AUROC) curve using the R package *ROCR* for each detection method and score type. Confidence intervals for other performance metrics including the AUROC were calculated using 100 bootstraps of the risk score distributions. Performance metrics followed the conventional definitions:

- AUROC was defined as the probability of a randomly chosen positive control score being greater than a randomly chosen negative control score.
- Sensitivity (i.e. power and true positive rate or TPR) was defined as the fraction of positive drug-events risks greater than a score threshold out of all positive drug-events.
- Positive predictive value (i.e. precision) was defined as the fraction of positive drug-events risks greater than a score threshold out of drug-events predicted as positive.
- Negative predictive value was defined as the fraction of negative drug-events risks at or less than a score threshold out of drug-events predicted as negative.

### Dynamics sensitivity analysis

We assessed the sensitivity of the ADE detection methods to detect drug event reporting dynamics within child development stages. We artificially removed, at 10% decrements, random drug reports at each child development stage separately. Removing drug reports lowers the rate of drug reporting while maintaining the event reporting rate. Specifically, at each stage, we determined the performance of each method and score type to detect (drug-event, stage, dynamic) score triples compared to the same negative control (drug-event, stage) score doubles at that same reduced stage. Sensitivity was assessed iteratively at the 10% decrements within each child development stage. We calculated performance metrics such as the above to quantify detection of drug event reporting dynamics for each method and score type.

### Real-world ADE validation

We applied the ADE detection methods on observed FAERS data for drug-event pairs within the pediatric drug-event reference standard from the Global Research in Pediatrics consortium [50]. A machine-readable dataset can be found at the ‘GRiP_pediatric_ADE-reference_set’ github repository (DOI: 10.5281/zenodo.4453379). We assigned drug-event pairs with epidemiological or mechanistic evidence in children (Control==‘C’) as the positive class (*N* = 26), and the cross-product of all drugs and events that were complementary to drug-event pairs in the reference set as the negative class (*N* = 123). We calculated the AUROC using the *ROCR* package in R and the true positive rate using the null statistic of each method as the prediction threshold.

## Supplementary Information

**Additional file 1.**

## Data Availability

**The FAERS data used in this study are publically available.** The datasets and code supporting the conclusions of this article are available in the ‘evaluating_ontogenic_ade_risk’ Github repository**, DOI:** 10.5281/zenodo.4945684.

## Abbreviations

ADE: Adverse drug event
FAERS: Food and Drug Administration Adverse Event Reporting System
GAM: Generalized additive model
PRR: Proportional reporting ratio
NICHD: National institute of child and human development
AUROC: Area under the receiver operating characteristic curve
TPR: True positive rate
ATC: Anatomical therapeutic class
DOI: Digital object identifier
