# Supplementary figures and images for: Evaluating risk detection methods to uncover ontogenic-mediated adverse drug effect mechanisms in children
Source: BioData Min. 2021 Jul 22;14:34. doi: 10.1186/s13040-021-00264-9 (PMC8296590; doi:10.1186/s13040-021-00264-9)

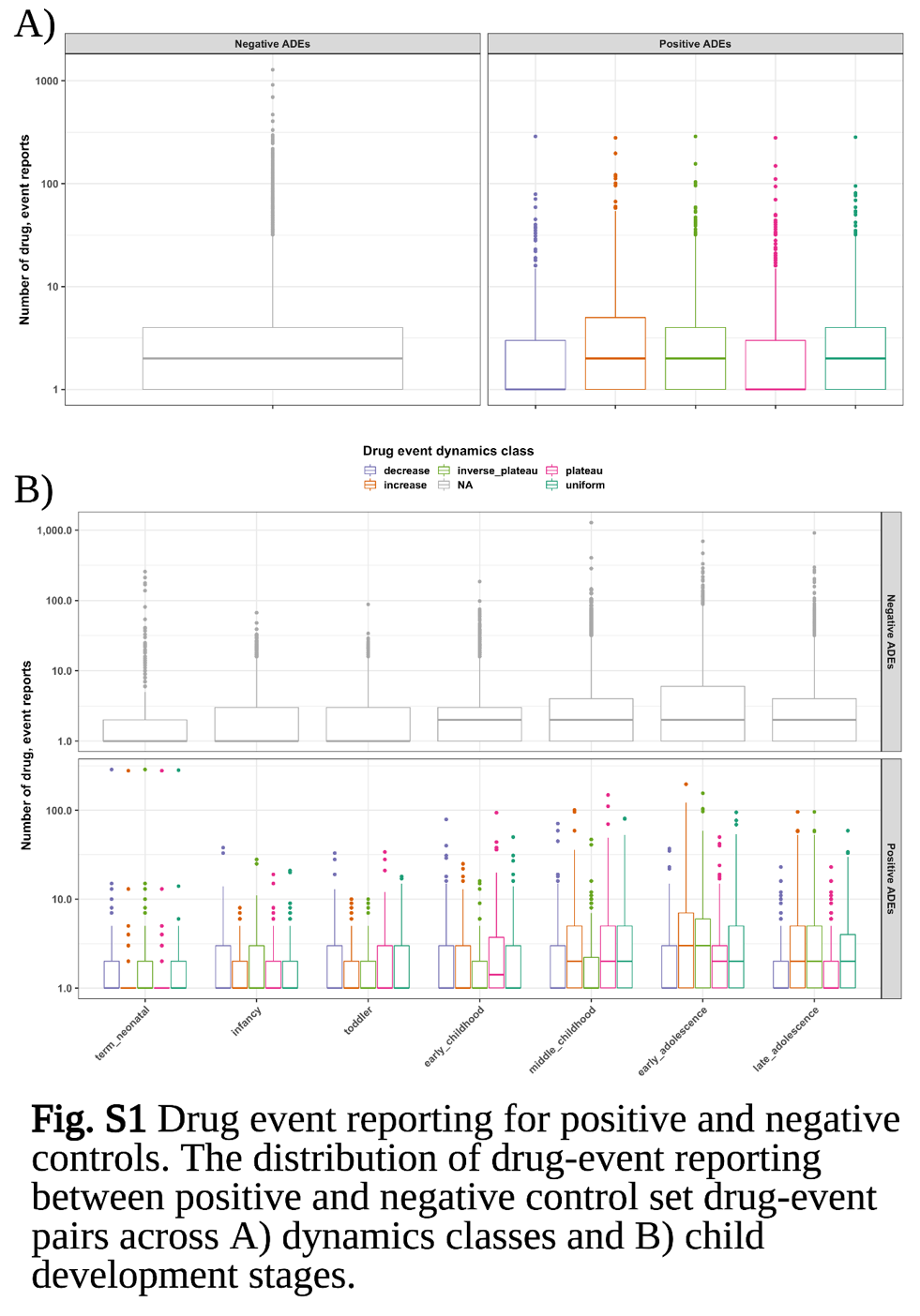


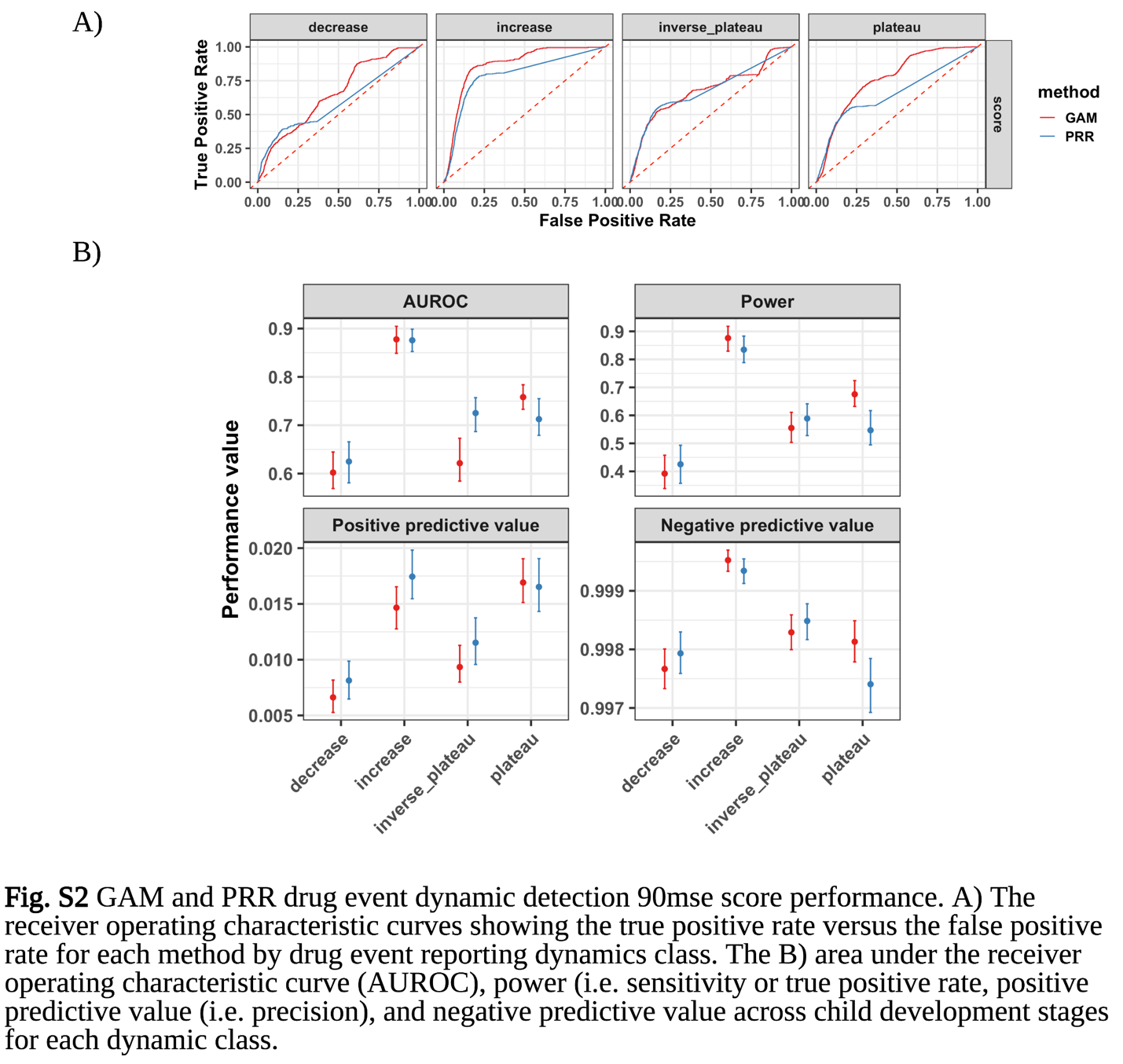


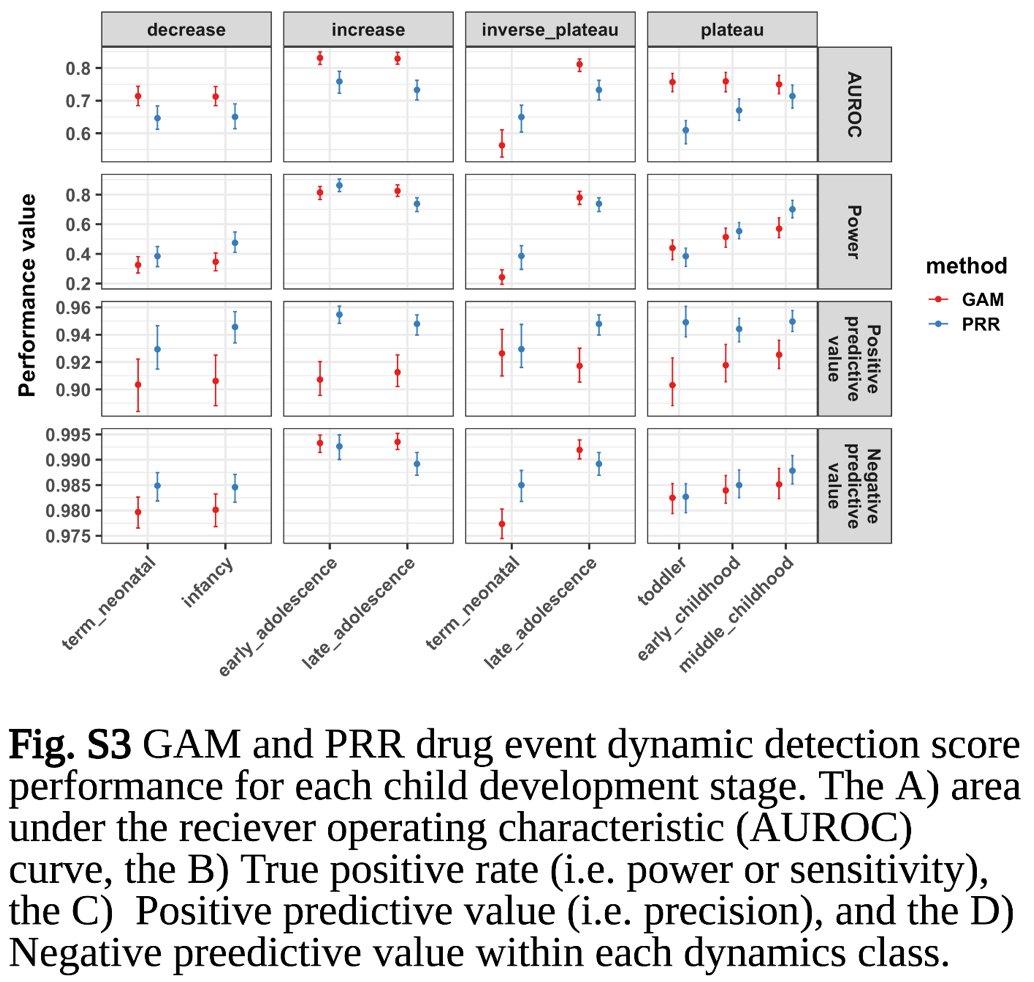

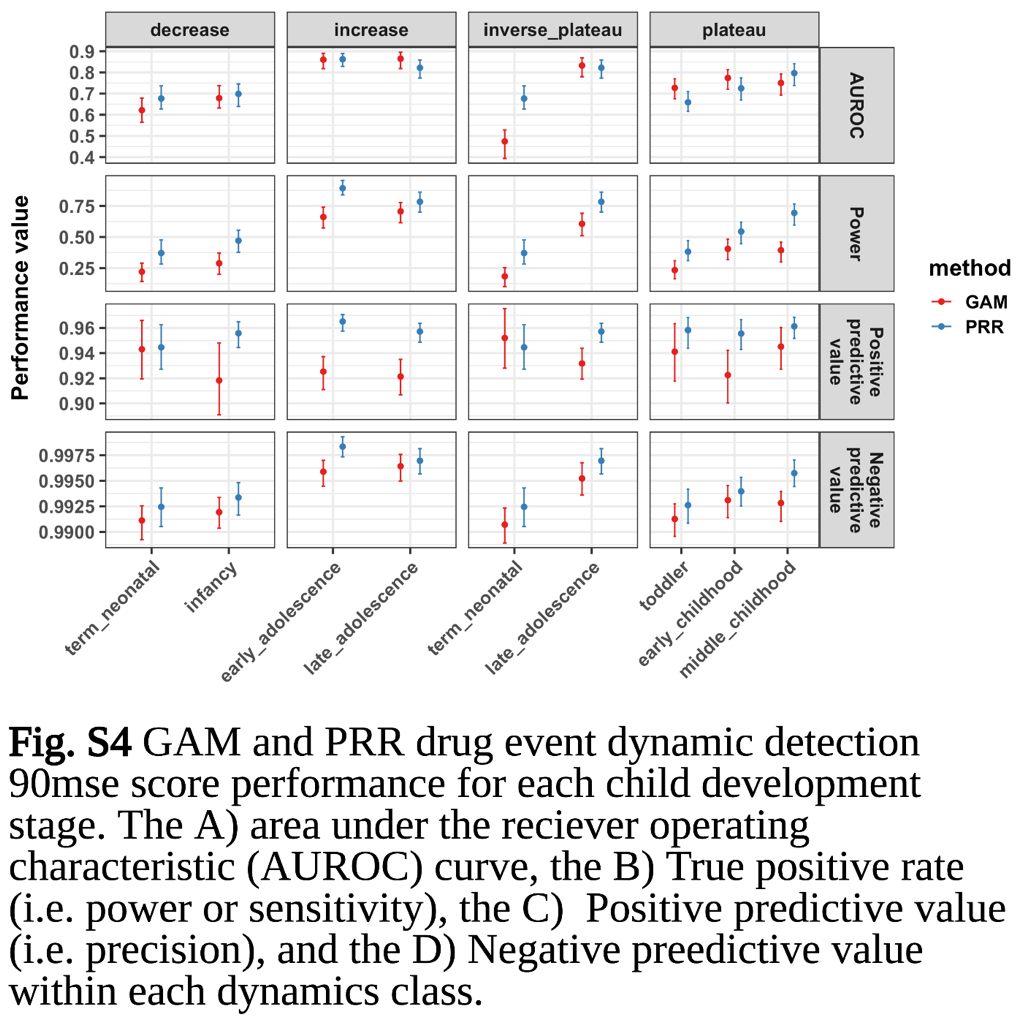


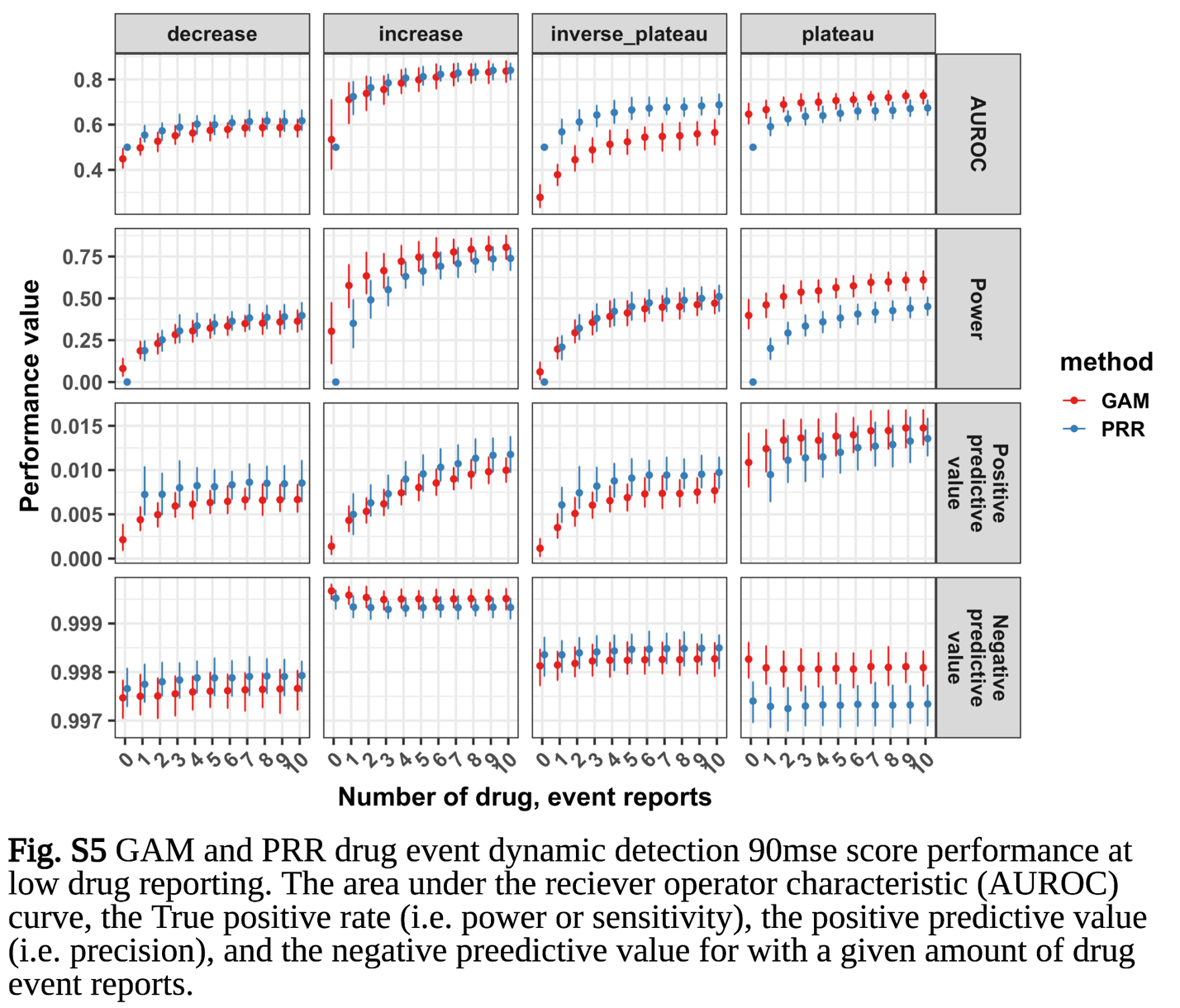


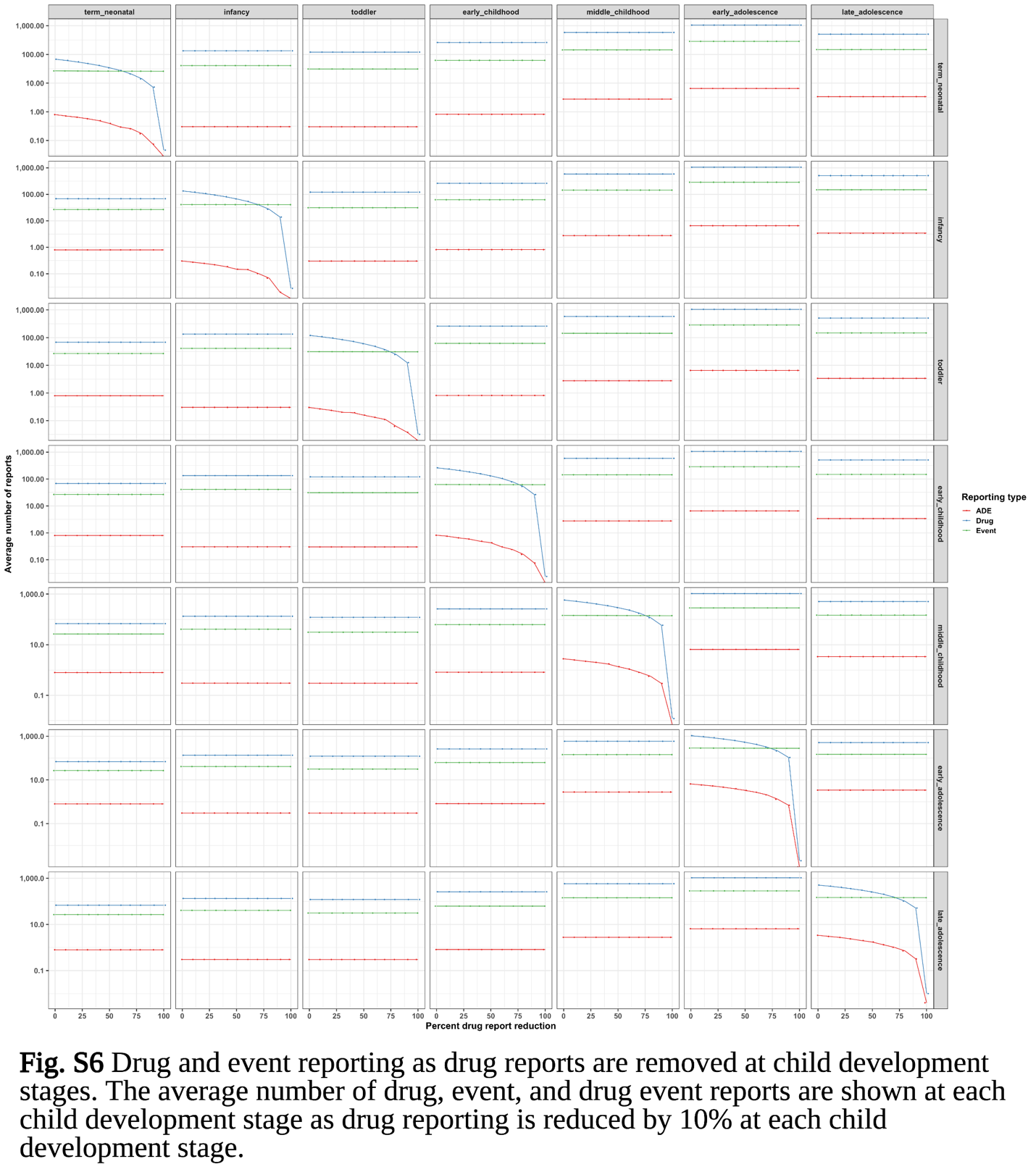


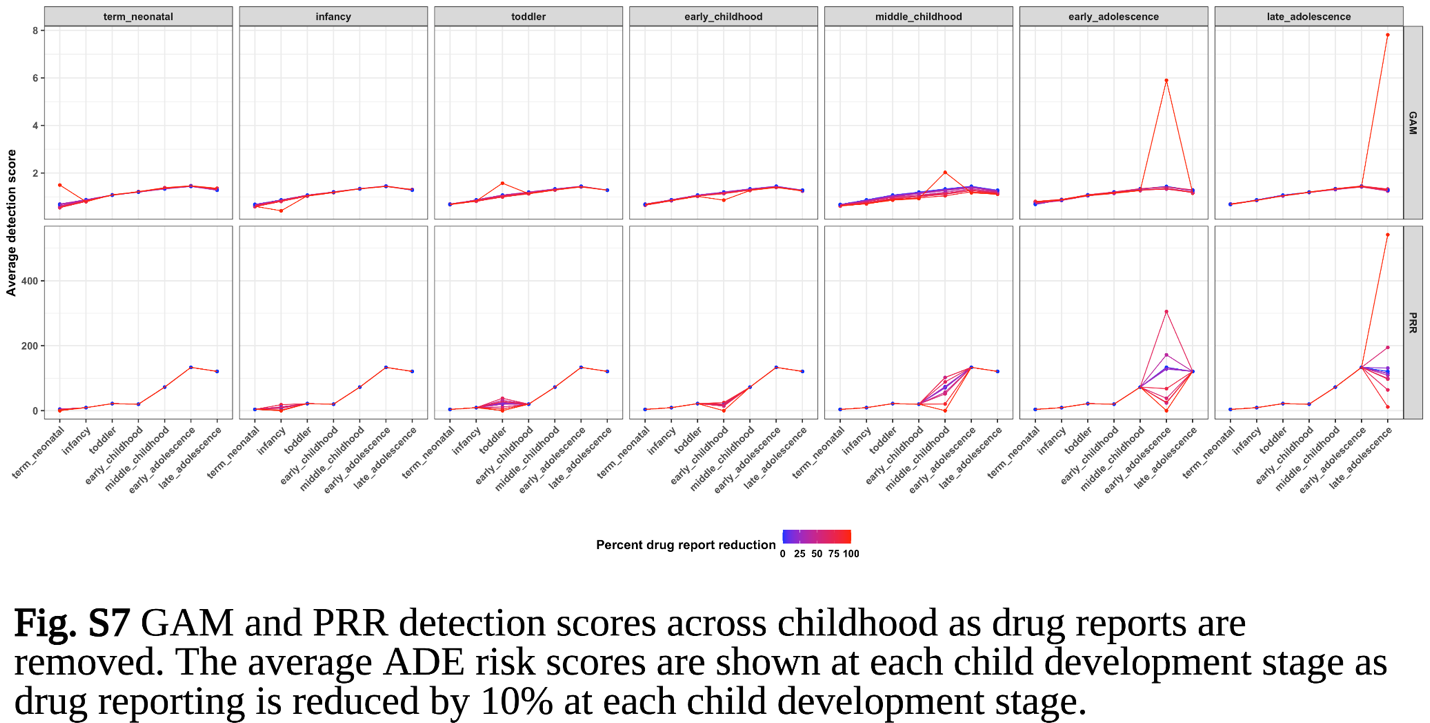


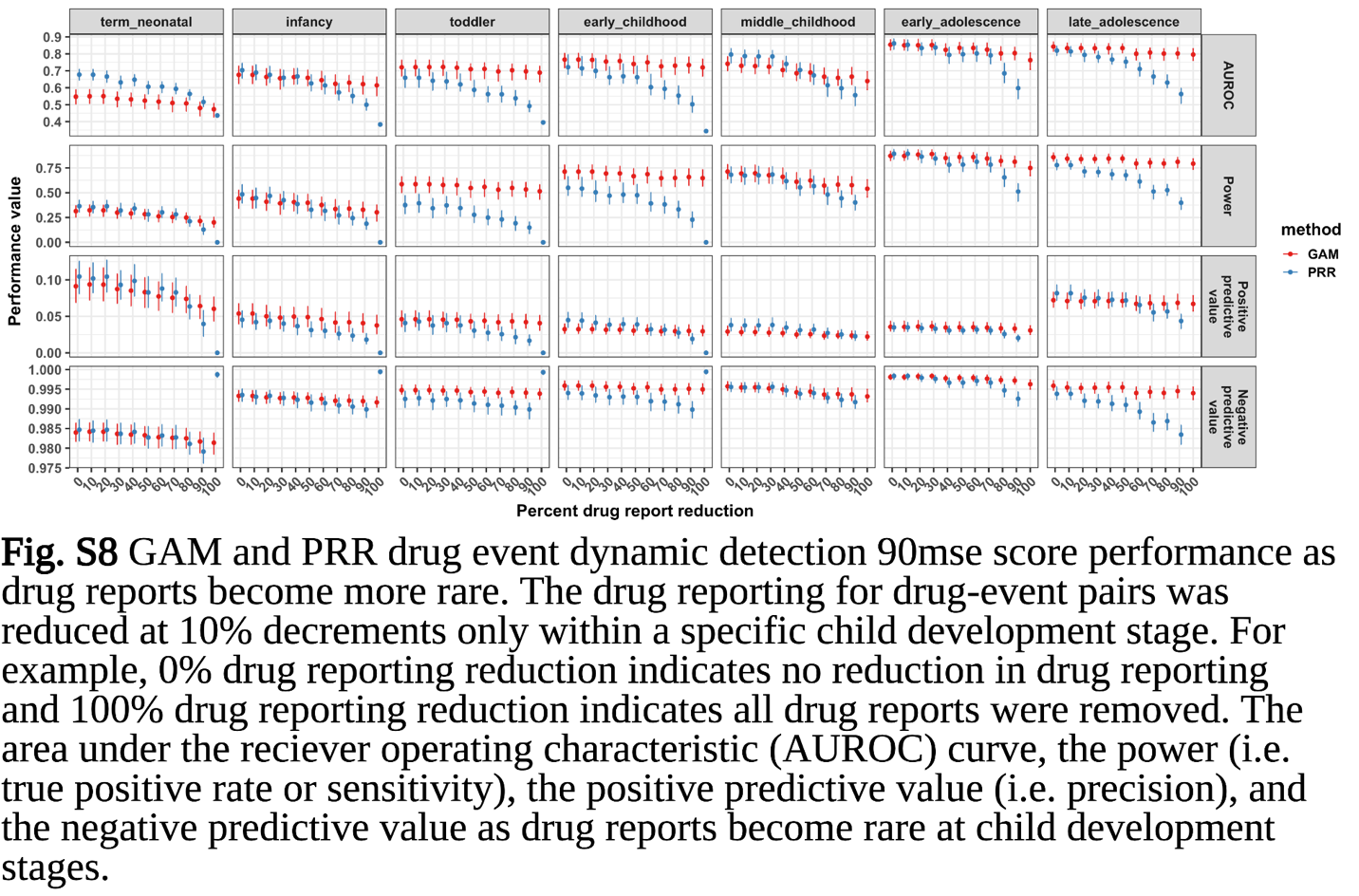


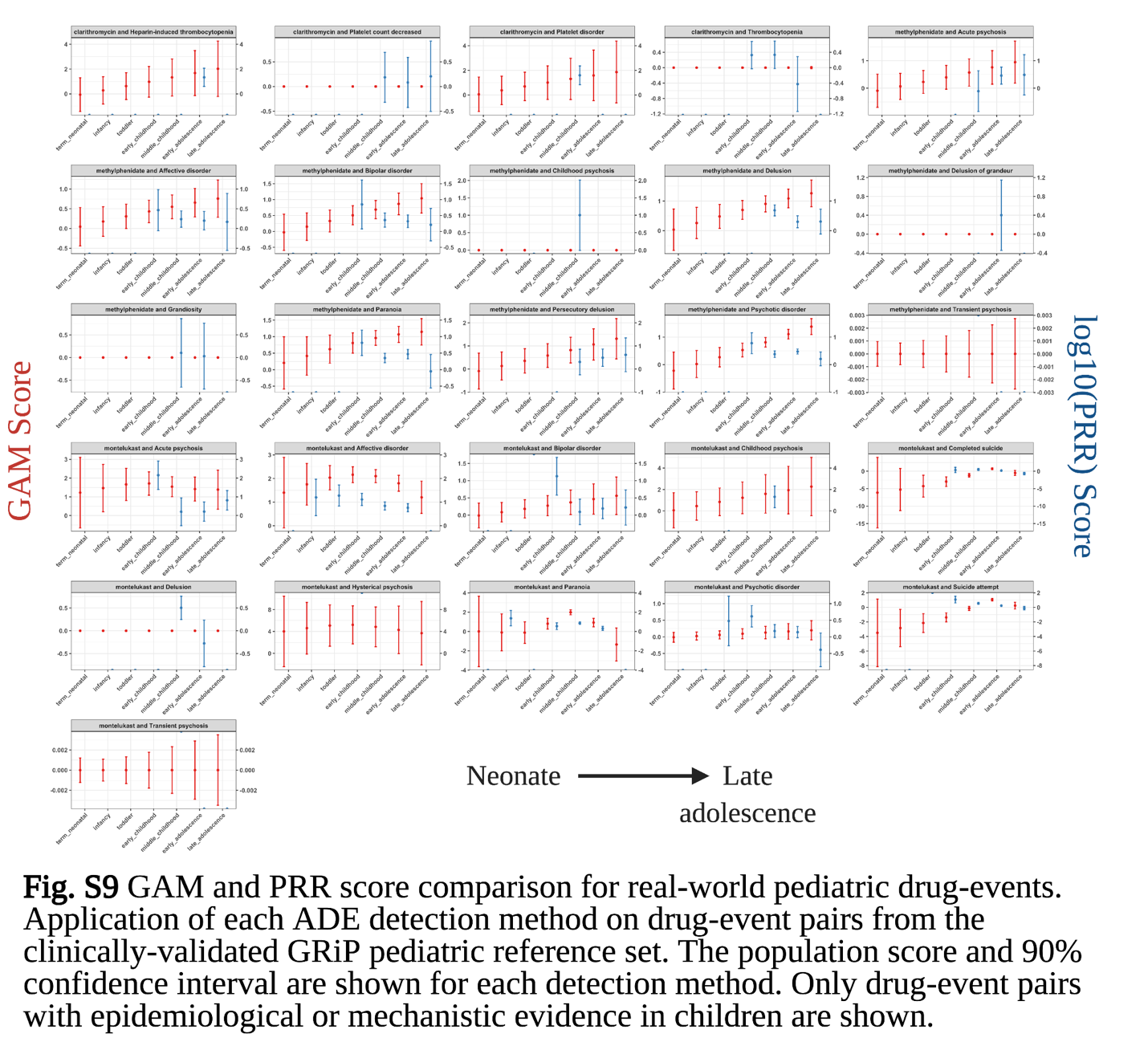


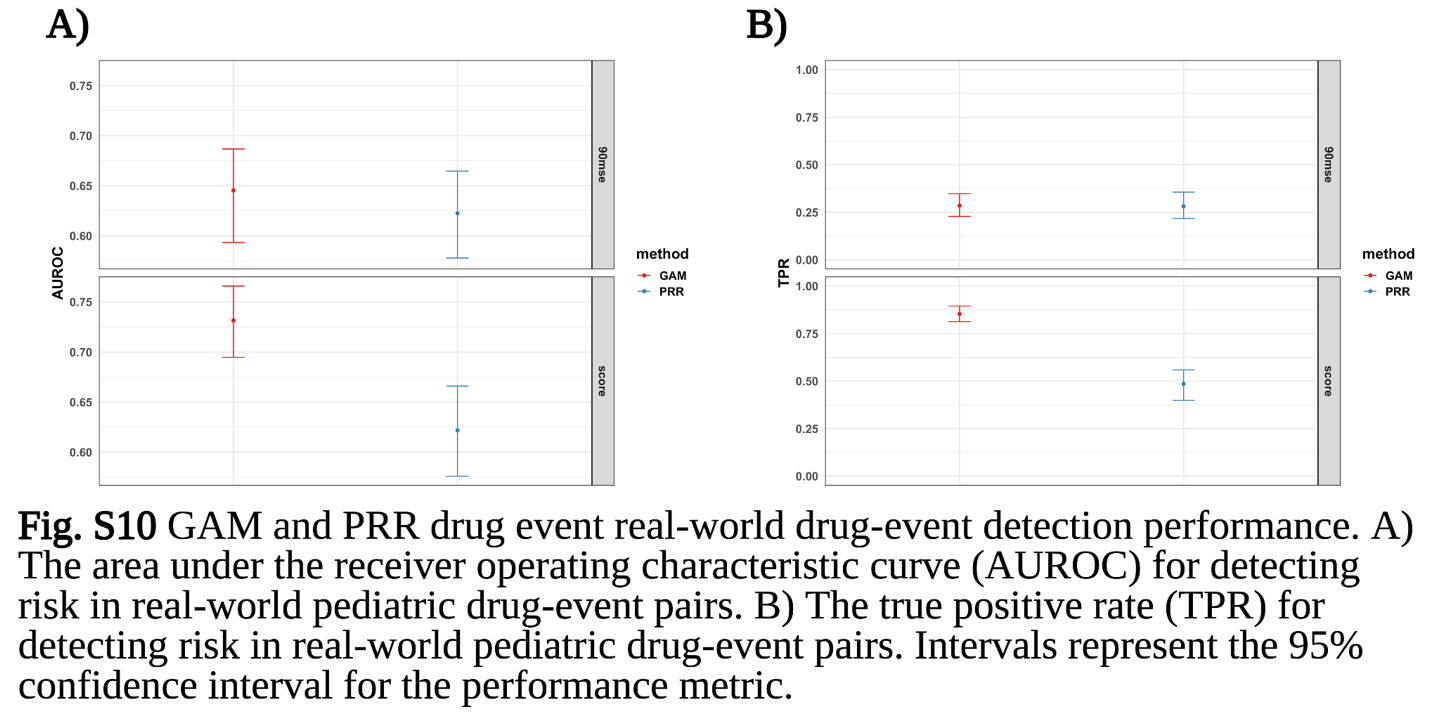


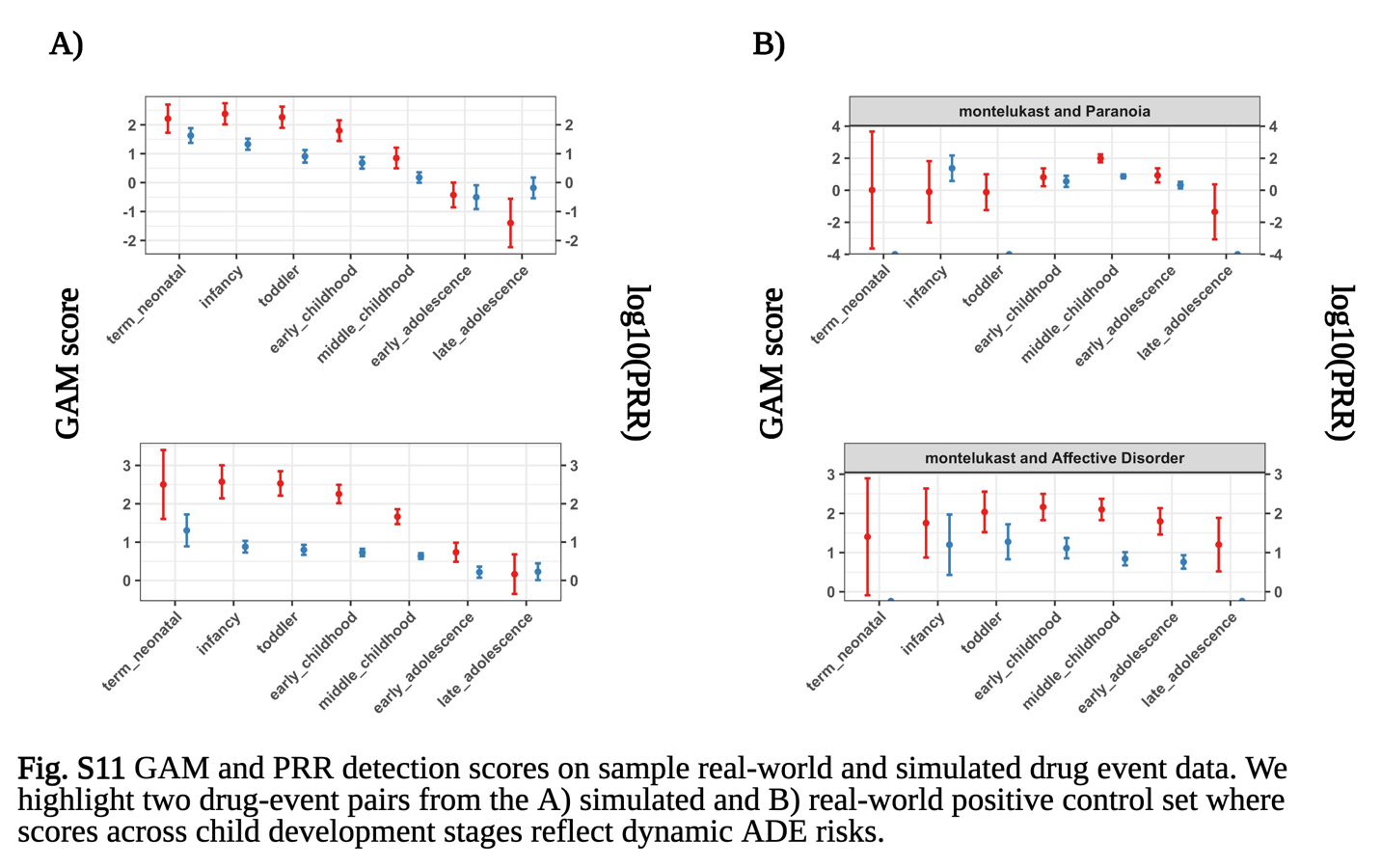


**
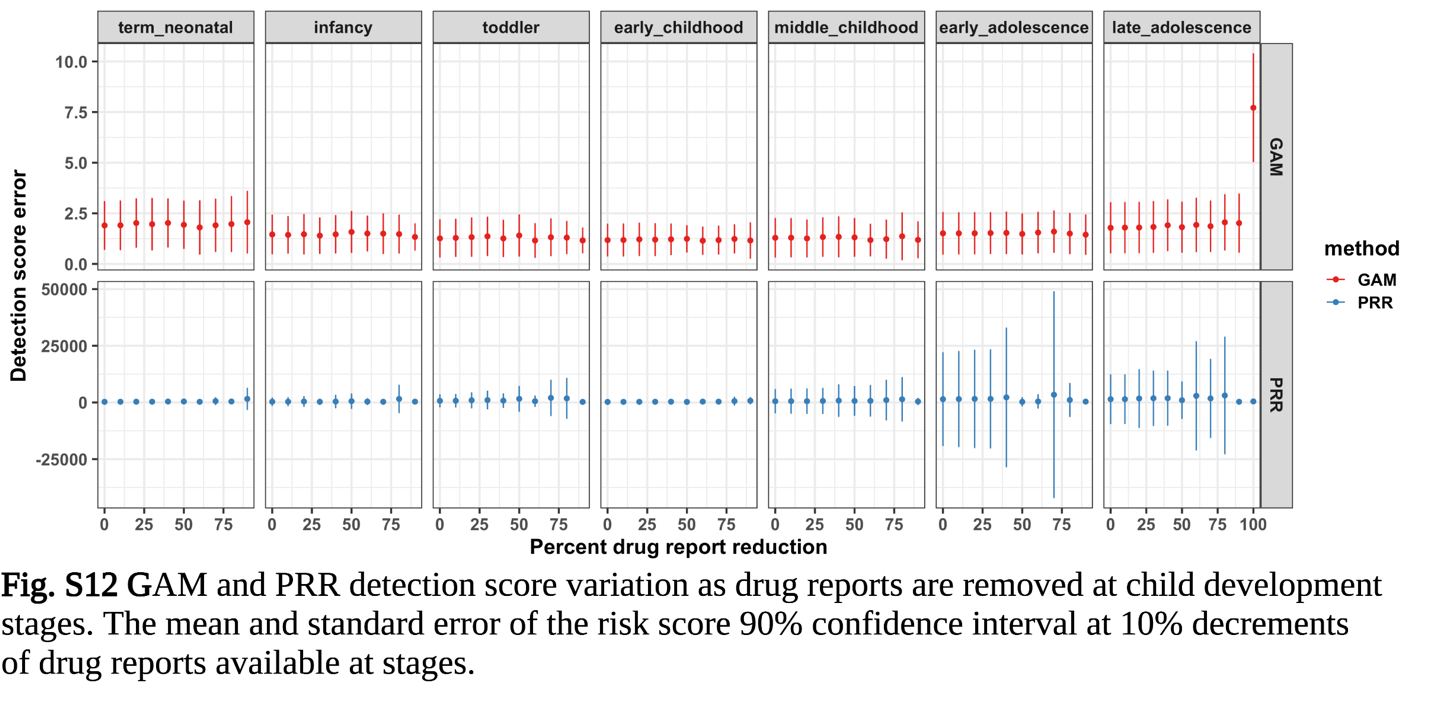

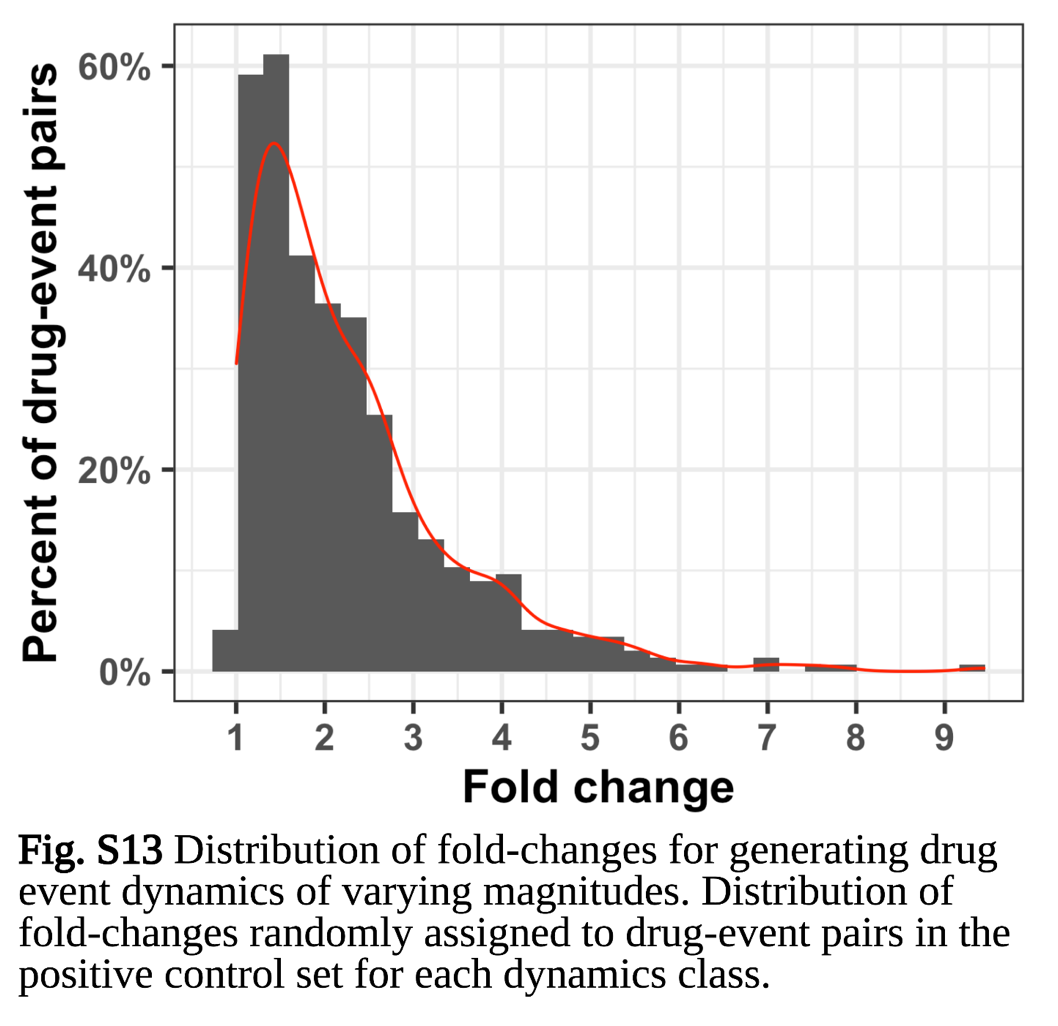
**


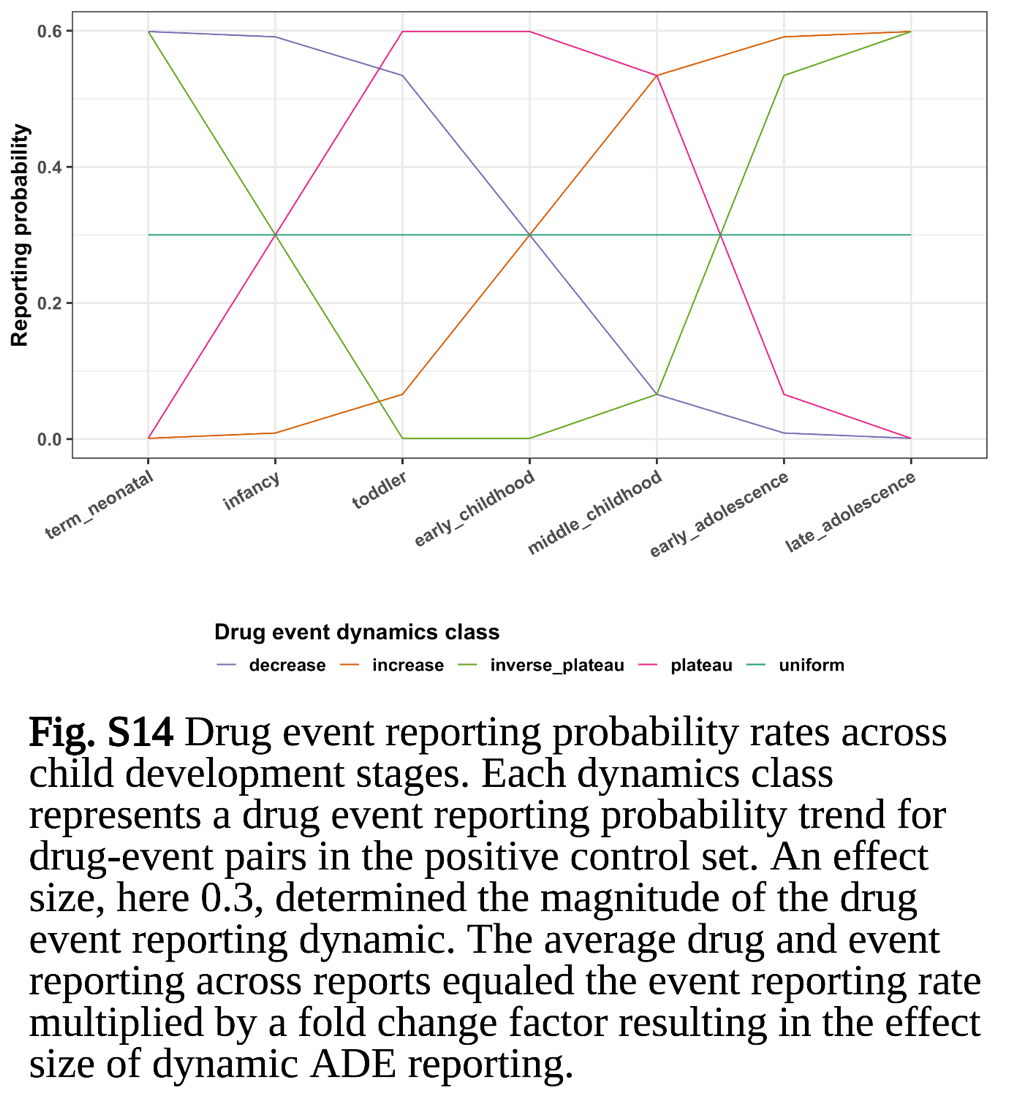


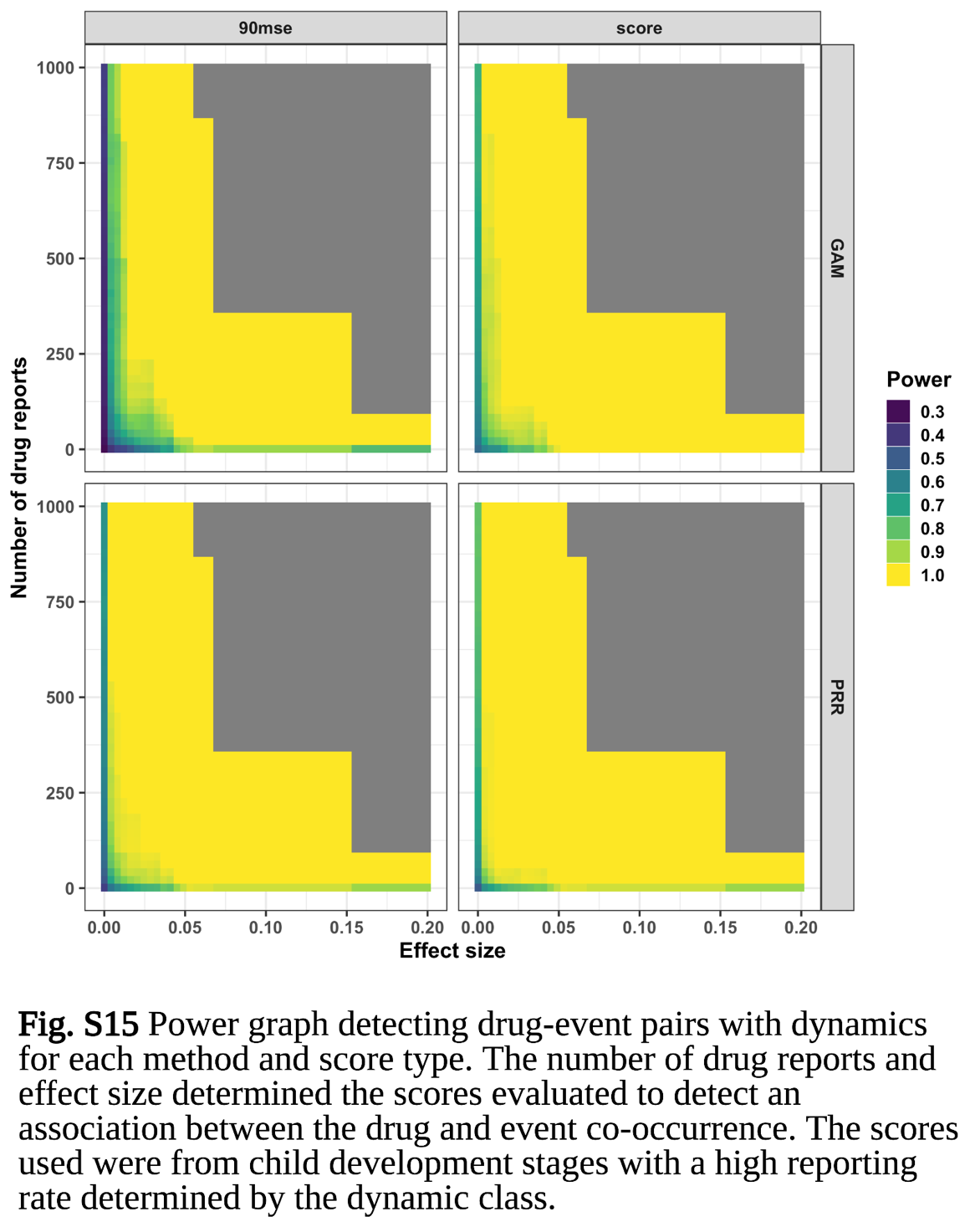


**
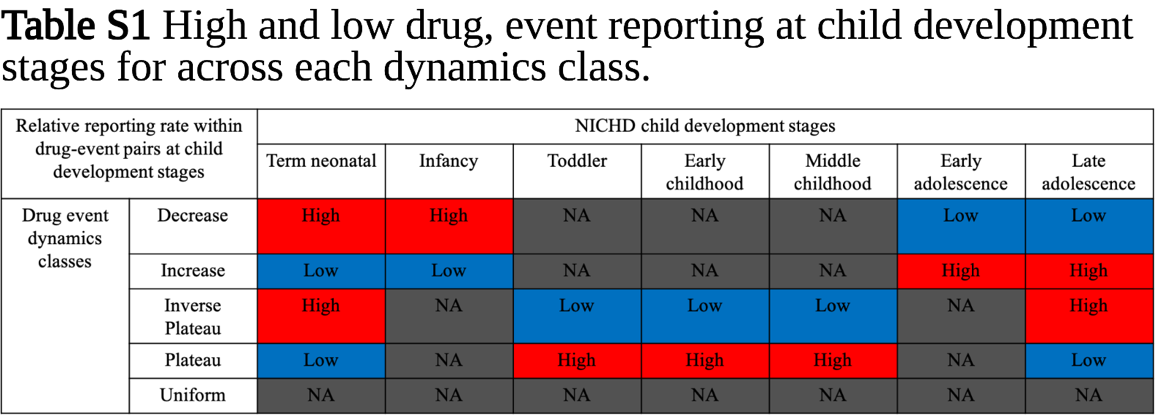
**

**
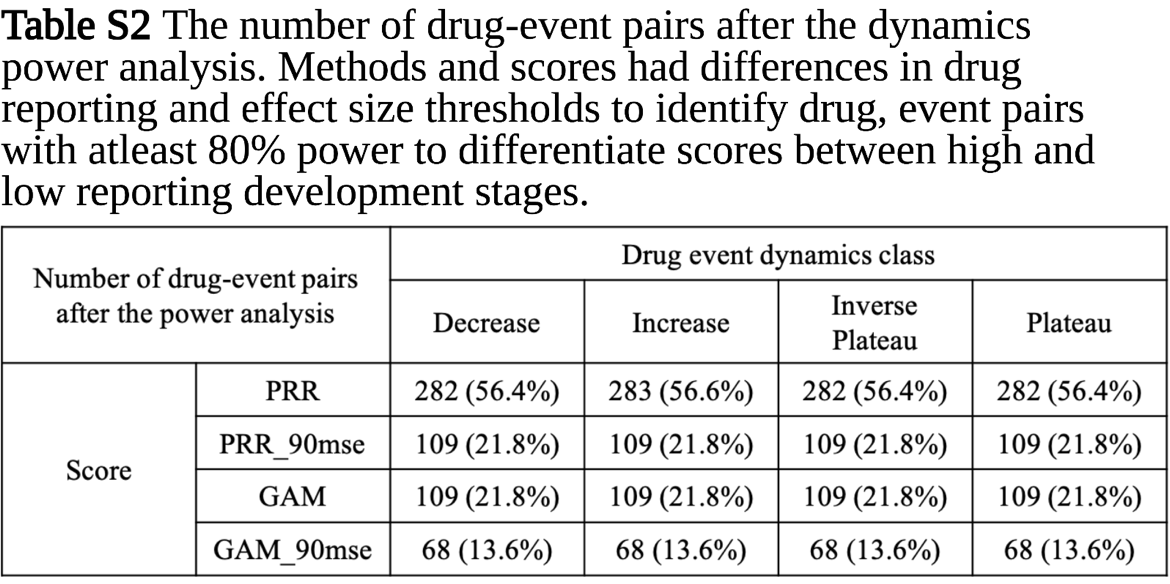
**

Supplement: Supplementary file 1 — Additional file 1. [file 13040_2021_264_MOESM1_ESM.docx]
